# Analysis of DNA Repair and Protection in the Tardigrade *Ramazzottius varieornatus* and *Hypsibius dujardini* after Exposure to UVC Radiation

**DOI:** 10.1371/journal.pone.0064793

**Published:** 2013-06-06

**Authors:** Daiki D. Horikawa, John Cumbers, Iori Sakakibara, Dana Rogoff, Stefan Leuko, Raechel Harnoto, Kazuharu Arakawa, Toshiaki Katayama, Takekazu Kunieda, Atsushi Toyoda, Asao Fujiyama, Lynn J. Rothschild

**Affiliations:** 1 Biospheric Science Branch, NASA Ames Research Center, Moffett Field, California, United States of America; 2 NASA Astrobiology Institute; 3 Department of Molecular Biology, Cell Biology and Biochemistry, Brown University, Providence, Rhode Island, United States of America; 4 INSERM U1016, Institut Cochin, Paris, France; 5 CNRS UMR 8104, Paris, France; 6 Université Paris Descartes, Sorbonne Paris Cité, Paris, France; 7 California Polytechnic State University, San Luis Obispo, California, United States of America; 8 Institute for Advanced Biosciences, Keio University, Fujisawa, Japan; 9 Human Genome Center, Institute of Medical Science, University of Tokyo, Tokyo, Japan; 10 Department of Biological Sciences, Graduate School of Science, University of Tokyo, Tokyo, Japan; 11 Center for Information Biology, National Institute of Genetics, Mishima, Shizuoka, Japan; 12 Principles of Informatics Research Division, National Institute of Informatics, Tokyo, Japan; Institute of Molecular Genetics IMG-CNR, Italy

## Abstract

Tardigrades inhabiting terrestrial environments exhibit extraordinary resistance to ionizing radiation and UV radiation although little is known about the mechanisms underlying the resistance. We found that the terrestrial tardigrade *Ramazzottius varieornatus* is able to tolerate massive doses of UVC irradiation by both being protected from forming UVC-induced thymine dimers in DNA in a desiccated, anhydrobiotic state as well as repairing the dimers that do form in the hydrated animals. In *R*. *varieornatus* accumulation of thymine dimers in DNA induced by irradiation with 2.5 kJ/m^2^ of UVC radiation disappeared 18 h after the exposure when the animals were exposed to fluorescent light but not in the dark. Much higher UV radiation tolerance was observed in desiccated anhydrobiotic *R*. *varieornatus* compared to hydrated specimens of this species. On the other hand, the freshwater tardigrade species *Hypsibius dujardini* that was used as control, showed much weaker tolerance to UVC radiation than *R. varieornatus*, and it did not contain a putative *phrA* gene sequence. The anhydrobiotes of *R*. *varieornatus* accumulated much less UVC-induced thymine dimers in DNA than hydrated one. It suggests that anhydrobiosis efficiently avoids DNA damage accumulation in *R*. *varieornatus* and confers better UV radiation tolerance on this species. Thus we propose that UV radiation tolerance in tardigrades is due to the both high capacities of DNA damage repair and DNA protection, a two-pronged survival strategy.

## Introduction

Tardigrades are invertebrate animals found in oceans, freshwater, and terrestrial environments. Under arid conditions, some tardigrade species inhabiting terrestrial environments lose more than 97 % of their body water content [1], [2], [3] and enter anhydrobiosis, an ametabolic dry state. Terrestrial tardigrades show extraordinary tolerance to ionizing radiation at the dose range of kGy [2]–[6]. Tardigrades also exhibit extraordinary resistance to UV radiation at doses of more than 10 kJ/m^2^ [7]. A small fraction of individuals (< 3%) of terrestrial tardigrade *Milnesium tardigradum* in the anhydrobiotic state survived a 10-day space flight exposure experiments at low Earth orbit even after exposure to 7577 kJ/m^2^ of UV radiation (116.5–400 nm wavelength) [8]. Since such massive exposure to UV radiation usually causes lethal DNA damage in organisms, tardigrades must bear enhanced capabilities for repairing heavily damaged DNA and/or protecting DNA against UV radiation. Yet, little is known concerning mechanisms underlying the tolerance of tardigrades to damage incurred by radiation. UVC radiation exposure usually causes formation of the major photoproduct thymine dimers, or thymine cyclobutane pyrimidine dimers (CPDs), in DNA [8]. As thymine dimer formation induced by UV irradiation leads the failure of priming DNA synthesis [9] and mutation [10], and therefore the dimer formation caused by UV radiation has been thought to have lethal effects on cells [11]. Thus, to examine thymine dimers formed in DNA by UV irradiation is of importance to estimate whether tardigrades cope with UV radiation exposure by protecting DNA against UV radiation or by repairing UV-induced DNA damage.

In the present study, we investigated UVC radiation-induced DNA damage as assessed by measurement of thymine dimers, in the terrestrial tardigrade species *Ramazzottius varieornatus* and the freshwater species *Hypsibius dujardini*. Resistance to UVC radiation as measured by survival and reproduction was much higher in hydrated *R*. *varieornatus* than hydrated *H*. *dujardini*, and light-activated DNA repair activity was detected only in *R*. *varieornatus* following UVC irradiation. In *R. varieornatus* individuals in the anhydrobiotic state has a higher survival and reproductive capability to UVC radiation than ones in the hydrated state, and in both states accumulated substantially fewer thymine dimers following UVC irradiation. We conclude that terrestrial tardigrades cope with massive UV irradiation by both effective DNA protection systems as well as DNA repair.

## Materials and Methods

### Tardigrade Strains and Culturing Conditions

In this study, two tardigrade strains were examined, *R. varieornatus* YOKOZUNA-1 and *H. dujardini* (Z151). The tardigrade *R*. *varieornatus* strain YOKOZUNA-1 was maintained in a culture system established previously [3] while the strain *H. dujardini* Z151 was purchased from Sciento Co. (Manchester, UK). Both strains were maintained in Petri dishes (90 mm in diameter) that contained distilled water with the green alga *Chlorococcum* sp. supplied by Sciento. Co.. Gels containing 1.5% bacto-agar were layered at the bottom of the dishes. The Petri dishes were covered with lids and placed in a moisture chamber to reduce water evaporation. The dishes were kept at 22°C in the dark. Animals were transferred to new culture dishes by a glass pipette every 1 to 2 weeks.

### Desiccation Tolerance

Capacity of desiccation tolerance was compared between *H. dujardini and R. varieornatus*. 19 to 21 animals suspended in 100 µl of distilled water were placed on a mesh filter (2 cm×2 cm, 15 µm of Mesh opening length, Product reference 03-15/10, SEFAR NITEX^®^, Switzerland) on a piece of filter paper (2×2 cm) in a plastic dish (35 mm in diameter). The animals were desiccated at 22°C for 5 d in a desiccator in which relative humidity was controlled at 33.8% with 85% glycerol solution at bottom. Relative humidity was controlled by using glycerol in water according to Johnson (1940) [12]. After desiccation the tardigrades were rehydrated with 2 ml of distilled water, and the number of active animals were counted 24 h after rehydration. Four replicates were used for each species in this experiment.

### Preparation of Hydrated and Desiccated Samples

To prepare hydrated samples, active *R*. *varieornatus* and *H*. *dujardini* which had been starved on a food-free culture dish for 20 h to eliminate food contamination, were picked up from the culture dish and washed in distilled water and placed, inside a drop of distilled water, on the center of a 1.5% bacto-agar Petri dish (90 mm in diameter). The excess water surrounding the animals was removed by a glass pipette and evaporated under laboratory conditions until water film covering the tardigrades is invisible. The animals were able to be hydrated and active under this condition at least up to 1 h since the animals were in touch with water through the moisture agar plate. The animals were then immediately irradiated with UVC (254 nm). For preparing desiccated samples, active *R*. *varieornatus* starved for 20 h in a food-free culture dish were desiccated at 22°C under 33.8% relative humidity in the same manner described above.

### Irradiation

Tardigrade samples were exposed to UVC radiation provided by a GE Germicidal lamp. The beam’s intensity was 0.57 mWs/cm^2^ at 254 nm, measured with a high resolution detector on a spectrometer (FMA2100 fitted with the PMA 2122 UVC Germicidal Detector, Solar Light Co., Inc.). The spectrum of the UV radiation emitted by the lamp was measured by Jazz Spectrometer Module (Ocean Optics, Dunedin, FL, USA) (Fig. S1). The UV dosage was calculated according to the formula 1 mW/cm^2^/sec = 1 mJ/cm^2^. The tardigrade samples were exposed to 0 to 20 kJ/m^2^ UVC radiation. Distance between the animals and the lamp was 4.2 cm. The maximum duration of UVC irradiation was 58 min 40 s for 20 kJ/m^2^. The animals were irradiated at 20 to 22°C under above 40% relative humidity. Irradiated samples were examined in subsequent experiments described as below.

### Post-Irradiation Survival and Reproduction

Survival and reproductive capability were evaluated for the post-exposure animals. Ten day old tardigrades were used for both *R*. *varieornatus* and *H. dujardini* experiments. In order to obtain the 10-d old individuals, in both the species eggs were transferred from culture dishes to an agar plate and incubated, and juveniles that hatched within 24 h were moved to a new culture dish on the day they hatched. Both hydrated and desiccated tardigrade samples were exposed to doses of 0, 2.5, 5, 10, and 20 kJ/m^2^ UVC radiation. At each dose 37 to 41 individuals (two replicates) were used except *R. varieornatus* in the anhydrobiotic state at 20 kJ/m^2^ (19 individuals in single group). After exposure, distilled water was added to both hydrated and desiccated samples, and the samples were transferred into culture dishes (35 mm in diameter) with distilled water and *Chlorococcum* sp.. The tardigrade culture was conducted as the same manner as described above. The animals in each culture dish were observed under a stereomicroscope over 30 days on a daily basis to record their survival, egg laying and hatching of laid eggs. Eggs deposited by the animals were removed to a small agar plate (35 mm in diameter) each day. Since developmental time of eggs is less than 10 d in *R. varieornatus* [3] and less than 5 d in *H. dujardini* [13], the isolated eggs were checked 7 and 14 d after oviposition, and hatched individuals were counted. The animals that extended their body with no motion were removed from the culture dishes. The number of eggs produced per animal was calculated by dividing the number of eggs by the total number of the tardigrades irradiated at each dose. For each dose group, the total number of eggs produced by animals and the total number of eggs that hatched was counted, and hatchability of eggs was calculated during the period of observation (30 d) after irradiation.

### Thymine Dimer Detection After UV Irradiation

To examine post-exposure thymine dimer formation in DNA in tardigrades, 50 to 60 *H*. *dujardini* and 80 to 100 *R*. *varieornatus* were used in each experiment. Both hydrated and desiccated samples were exposed to 0, 0.5, 5, and 20 kJ/m^2^ of UV radiation. Three replicates were used in this experiment. Some of these doses used here were different from those used to evaluate tolerance of tardigrades because it was expected that the difference in the number of DNA lesions among doses would be more clear if dose interval was wider than that used in the previous experiment. Immediately after UVC exposure, distilled water was added to the all kinds of samples, and the samples were transferred to a 1.5 ml microfuge tube with distilled water. The microtube was centrifuged briefly and supernatant in the tube was removed by a glass pipette. DNA was extracted from the exposed samples using the Nucleospin DNA extraction kit (Marcherey-Nagel GmbH & Co. KG, Düren, Germany). Each sample’s DNA was suspended in 40 µl of elution buffer in a microfuge tube.

The amount of DNA in each sample was quantified by comparison with that of a commercial standard DNA marker GelPilot plus 1kb (Qiagen, Valencia, CA, USA) in which the DNA amount is known. Aliquots (0.89, 1.33, and 3.00 ng) of the standard DNA marker and 2 or 3 µl of the sample DNA were loaded on 1% agarose gels containing 30 ml of TE buffer and 0.6 µl of ethidium bromide, and electrophoresis was conducted at 100 V for 5 min at room temperature. After electrophoresis, the gel was scanned with a Typhoon Trio Scanner (GE Healthcare, Piscataway, NJ, USA). Signal intensity was measured for each DNA band, and each sample’s DNA amount was quantified using the software ImageQuant TL (GE Healthcare).

Thymine dimers were detected according to the protocol of Sinha et al. (2001) [14] with some modifications. Ten ng of DNA from each sample were used for thymine dimer detection. The DNA samples were denatured by adding 1/10 volume 1 M NaOH, and then incubated at 80°C for 30 min. Samples were blotted onto an Amersham Hybond^TM^-LFP membrane with the Minifold^®^ Dot-Blot Manifold (Whatman Inc, NJ, USA) and the membrane was desiccated for 30 min at 80°C. Non-specific binding sites on the membrane were blocked by incubating the membrane in PBS-T buffer with 5% ECL advanced^TM^ blocking reagent (GE Healthcare) for 1 h at room temperature. The membrane was incubated at 37°C for 2 h with the primary antibody (mouse anti-thymine dimer, Kamiya Biomedical; 1∶3000 dilution in PBS-T). Then, the membrane was washed three times with PBS-T buffer and incubated for 1 h with the secondary antibody (sheep anti-mouse IgG with Cy3, Kamiya Biomedical; 1∶1000 diluted in PBS-T with 5% BSA). The membrane was washed again and thymine dimers were visualized with the Typhoon Trio Scanner, and the intensity of each dot was measured by the ImageQuant TL software. The pUC 19 plasmid DNA, which contained 5.17×10^4^ thymine dimers per 1 Mb was used as standard, and thus the frequency of thymine dimer formation in each DNA sample was calculated comparing intensity of the samples to those of the standards. The pUC 19 plasmid DNA which contains 5.17×10^4^ thymine dimers was used as molecular standard in this experiment.

### DNA Repair Detection

Hydrated samples of *R*. *varieornatus* and *H. dujardini* that had been starved for 20 h were irradiated with 2.5 kJ/m^2^ of UVC radiation at which dose survival of *R. varieornatus* was confirmed. 80 to 100 *R*. *varieornatus* and 50 to 60 *H*. *dujardini* were used in each experiment, and three independent experiments were carried out. After irradiation, the irradiated animals were transferred onto a food-free 1.5% agar plate (for the animals analyzed at 18 h after irradiation) or a 1% agar plate with *Chlolococcum* sp. (for the animals analyzed at 112 h after irradiation). DNA was extracted from the irradiated animals at 0, 18, and 112 h after irradiation and the number of thymine dimers in DNA from the animals in each group was quantified as described above. To evaluate whether light-dependent DNA repair occurred, one group of each species was kept in the dark, and the other was continuously illuminated by fluorescent light at room temperature. The animals in the latter group were transferred from the food-containing culture dish to a food-free culture dish at 94 h after irradiation, and the animals were starved for 18 h before DNA extraction. The pUC 19 plasmid DNA with 5.17×10^4^ thymine dimers was used as molecular standard.

### Bioinformatics

Tardigrades belong to the invertebrate superclade *Ecdysozoa* as do *Caenorhabditis elegans* and *Drosophila melanogaster.* A tardigrade photorepair gene has not been identified as of yet so the photorepair protein gene *phrA* from *D. melanogaster* was used (Accession: NM_078929) to search the *R. varieornatus* genome for homologous genes. BlastP [15] was performed with the amino acid sequence against the in house protein database predicted from *R. varieornatus* genome to search for homologues of the *Drosophila melanogaster* photorepair gene *phrA*. To search the homologous gene from *H. dujardini*, their EST sequences (http://www.ncbi.nlm.nih.gov/genomeprj/20353) were mapped to the genome of *R. varieornatus* by using Exogenerate program [16]. Protein domains of the *phrA* genes were predicted using Pfam [17], and amino acid sequence alignment was performed using MAFFT version 6 [18].

### PCR

Primers were designed to amplify part of the putative *phrA* gene: PhrA-tar-1-F: 5′ CGC TCC TCT GCG GCA CTT CC 3′ and PhrA-tar-1-R: 5′ ATC TGC GCG GCG TTC CAC AA 3′ using Geneious [19] and Primer3 [20]. DNA extractions were performed on *R. varieornatus* and *H. dujardini* as described above. Temperature gradient PCR was performed in a range of 58 to 72°C on each species under various reaction conditions in order to optimize PCR amplification. Platinum taq (Invitrogen, Carlsbad, CA, USA), GoTaq (Promega, Madison, WI, USA) and a range of MgCl concentration (1.5 to 6 mM) were used. Amplicons were cloned into the pGEM-T vector (Promega Inc. Madison, WI, USA), and colonies were picked and sequenced to three times coverage (Elim, Hayward, CA, USA).

### RNA Extraction and qPCR

In order to know if PhrA is involved in post-irradiation DNA repair, expression level of *phrA* gene was evaluated at 0, 18, and 112 h after exposure to 2.5 kJ/m^2^ of UV radiation. RNA was extracted from *R. varieornatus* by using TRIzol reagent (Invitrogen, Carlsbad, CA, USA). Around 250 individuals of tardigrades were transferred into a 1.5 ml microtube, and 100 µl TRIzol was added into the tube. After the animals were homogenized by a plastic pestle in the tube at room temperature, 900 µl TRIzol was added. 200 µl of chloroform was added into the tube. The tube was shaken by hand for 15 s and incubated for 3 min at room temperature. 500 µl of the transparent aqueous upper phase containing RNA was transferred into a new microtube, 500 µl of 70% ethanol was added, and the solution was mixed. Then RNA was washed and eluted by using PureLink RNA Mini Kit (Invitrogen). After RNA was eluted in 30 µl of distilled water, DNA was digested by DNase I using TURBO DNA-free Kit (Applied Biosystems). RNA amount was quantified by measuring UV absorbance at 260 nm.

First strand cDNA was obtained by reverse transcription (RT) from purified RNA in each sample using High Capacity cDNA Reverse Transcription Kits (Applied Biosystems). 100 ng of RNA was incubated with 4 mM dNTP mix and RT random primers at 25°C for 10 min, 37°C for 120 min, and 85°C for 5 min. After incubation the samples were put on ice.

Real-time PCR reaction was performed using LightCycler® 480 (Roche). The cDNA template yielded in the previous step was diluted by 4 fold, and 5 µl of diluted cDNA template was mixed with 500 nM of each oligonucleotide primer and 5 µl of LightCycler® 480 SYBR Green I Master (Roche). The oligonucletide sequences are as follows; elongation factor 1 alpha (EF1a) F, 5′- GGA GAC TGC CTC TTT CAA CG -3′; EF1aR 5′- ATC CAA GAC GGG TGT GTA GC -3′; PhraF, 5′- TTT TCG TGA TGA AGC TGT GC -3′; PhraR, 5′- TCC TCC TGG GTT TCA GAT TG -3′. PCR was performed as 5 min pre-incubation of 95°C, subsequently, 45 cycles of amplification, 10 s at 95°C, 10 s at 60°C 10 s at 72°C. Water without cDNA template was used as a negative control. Diluted cDNAs mix of all samples was used as a standard curve to calculate relative amounts of *EF1a* and *phrA.* The relative *phrA* expression level was normalized by *EF1a* expression level. Three or four independent experiments were performed in the Real-time PCR amplification.

### Statistics

Egg hatchability between groups in the hydrated *R. varieornatus* was compared by Chi-square test. Linear regression analysis was utilized for detection of correlation between UV radiation dose and egg hatchability in the anhydrobiotic state. For detection of correlation between UV radiation dose and the frequency of thymine dimer formation, nonlinear regression (curve fit) analysis was performed. The frequency of thymine dimer formation among groups was compared by Two-way ANOVA. Expression levels of *phrA* among groups were compared by One-way ANOVA. All statistic analysis was performed using software Prism ver. 5.0d for Mac OS X.

## Results

### Survival and Reproduction

**Figure 1:**
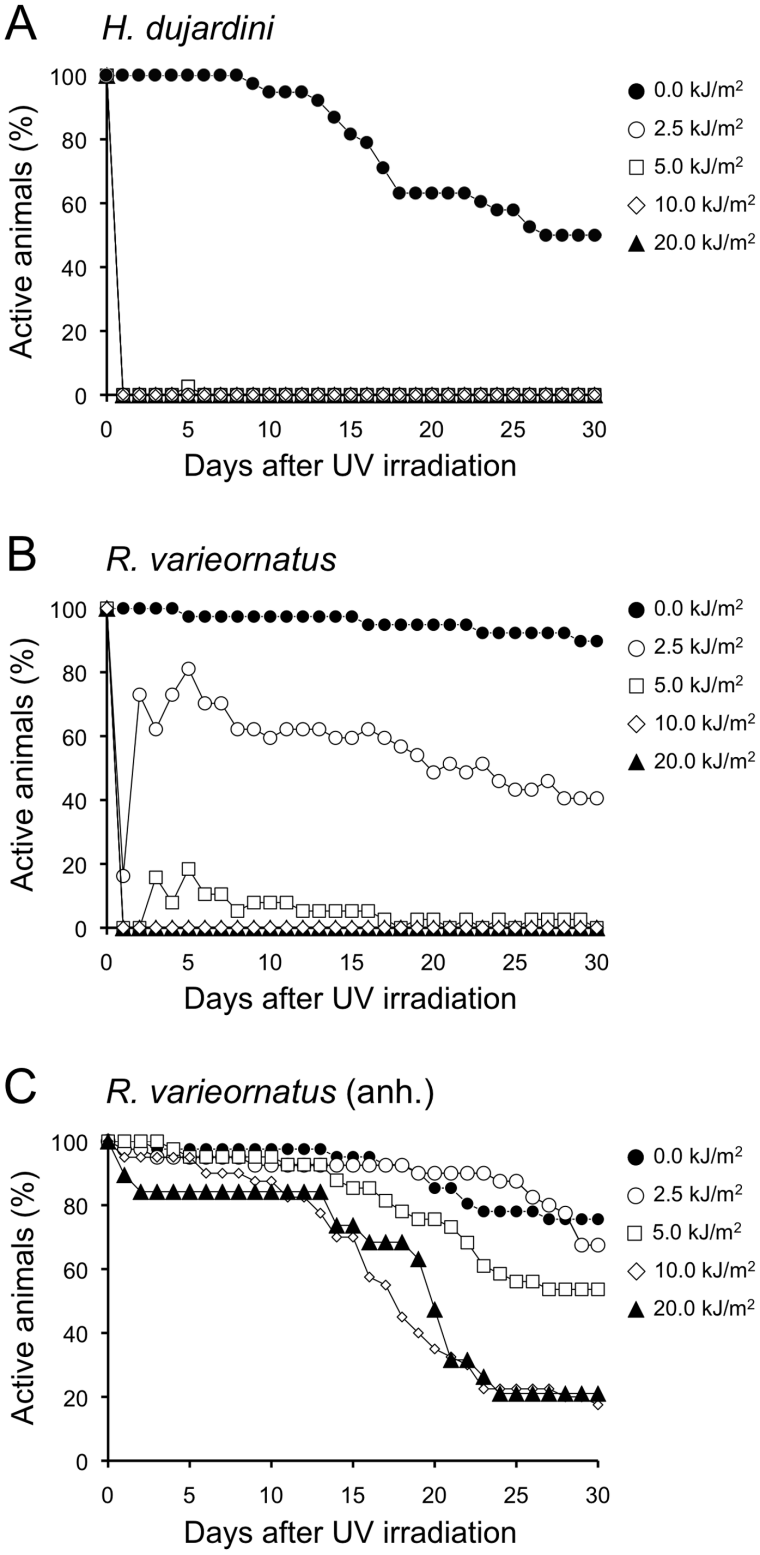
Time courses of percentage of active tardigrades after exposure to UVC radiation. (A) *Hypsibius dujardini* and (B) *Ramazzottius varieornatus* when exposing to UVC radiation in hydrated state, and (C) *R. varieornatus* when in anhydrobiotic state.

**Figure 2:**
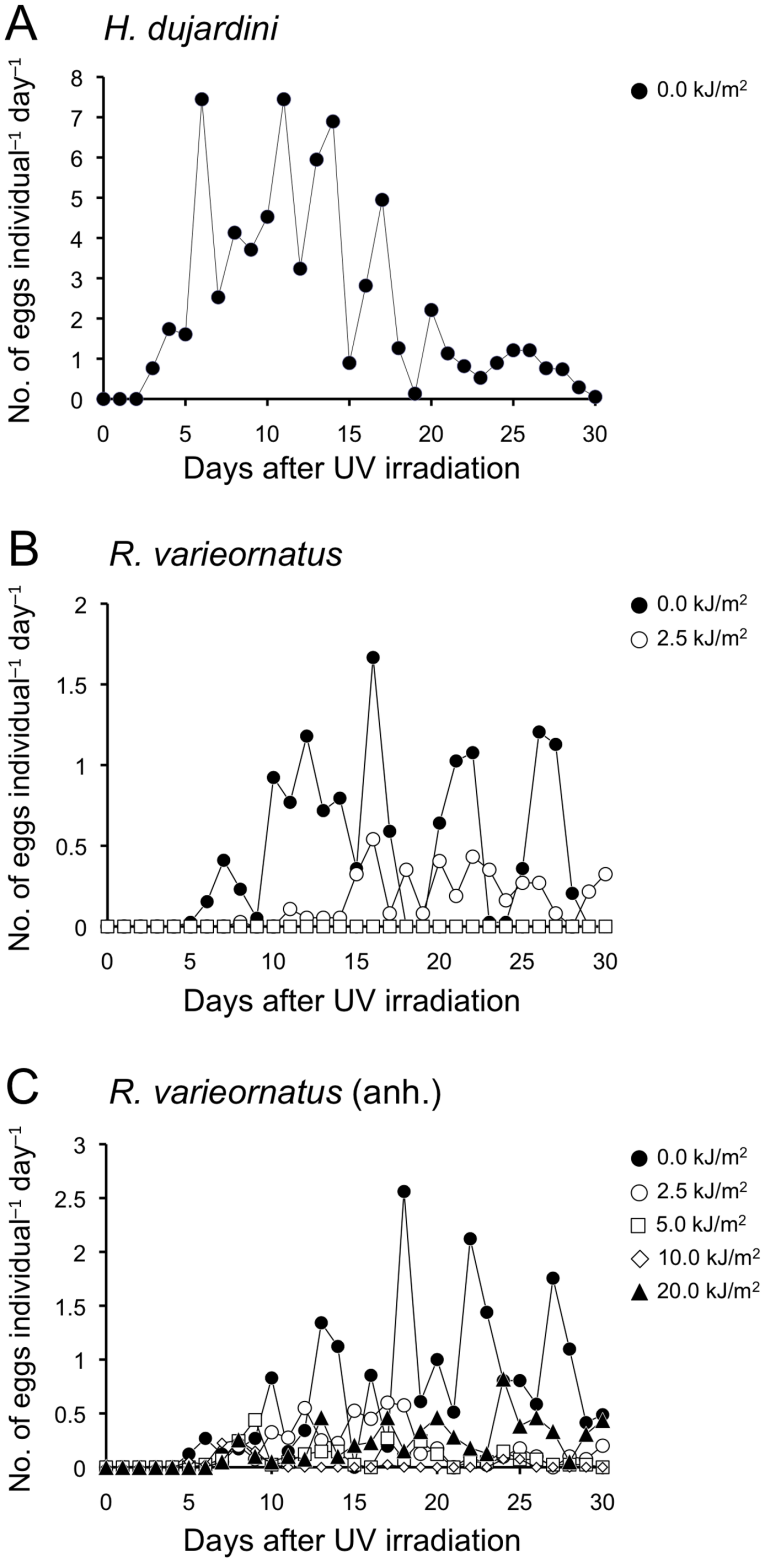
Time course of the number of eggs deposited per an irradiated adult after exposure to UVC radiation. (A) *Hypsibius dujardini* and (B) *Ramazzottius varieornatus* when exposing to UVC radiation in hydrated state, and (C) *R. varieornatus* when in anhydrobiotic state.

In non-irradiated groups the number of active individuals and eggs produced decreased more rapidly in *H. dujardini* than *R. varieornatus* (Fig. 1ABC, Fig. 2ABC), indicating that *H. dujardini* has shorter life span and reproduction period compared with *R. varieornatus*. Hydrated *R*. *varieornatus* exhibited higher survival ability than *H. dujardini* after exposed to UVC radiation (Fig. 1AB). UVC irradiation with over 2.5 kJ/m^2^ caused the instant death of *H. dujardini*, whereas 81.1% of *R. varieornatus* specimens were active 5 days after irradiation with this tested dose. However, none of the *R. varieornatus* individuals survived exposure to UVC radiation over 10 kJ/m^2^. In contrast to the hydrated *R. varieornatus*, anhydrobiotes of this species showed much higher survival following UVC irradiation (Fig. 1C). *R. varieornatus* irradiated in the anhydrobiotic state survived exposure to doses of UVC radiation tested. Over 80% of the animals exposed to even 20 kJ/m^2^ of UVC radiation were active at 10 d after irradiation.

Table 1 exhibits post-UVC irradiation reproduction activity of tardigrades for each dose group. *H. dujardini* produced 2655 eggs in total with hatchability of 0.83, but no eggs were obtained from irradiated groups as all of individuals irradiated died. *R. vaireornatus* irradiated in the hydrated state with 2.5 kJ/m^2^ of UVC radiation left 162 eggs, but none of survivors of *R. varieornatus* irradiated with 5 kJ/m^2^ produced eggs. In *R. varieornatus* irradiated in the anhydrobiotic state both egg and progeny production were confirmed at all doses examined, although irradiation had significant negative effects on reproductive ability. Egg production was confirmed even 25 d after irradiation in *R*. *varieornatus* irradiated with 2.5 kJ/m^2^ of UVC radiation in the hydrated state and all doses in the anhydrobiotic state (Fig. 2). Hatchability of eggs produced from *R*. *varieornatus* irradiated in the hydrated state was 0.90 at a dosage of 2.5 kJ/m^2^, while that from specimens irradiated in the anhydrobiotic state varied from 0.76 to 0.89. There was no significant difference in egg hatchability between groups of 0 and 2.5 kJ/m^2^ in the hydrated *R. varieornatus* (chi-square test: *P* = 0.936). In addition, no significant correlation between UV radiation dose and egg hatchability in the anhydrobiotic state was detected (linear regression analysis: *r* ^2^ = 6.5×10^–3^, *P* = 0.9).

**Table 1:** Capability of reproduction after UVC irradiation in *Hypsibius dujardini* and *Ramazzottius varieornatus*. n/a means no data available.

Species	Dose, kJ/m^2^	Individuals irradiated, n	Total eggs, n	Total eggs hatched, n	Hatchability
*H. dujardini*	0	38	2655	2199	0.83
	2.5	39	0	0	n/a
	5.0	38	0	0	n/a
	10.0	40	0	0	n/a
	20.0	40	0	0	n/a
*R. varieornatus*	0	39	529	465	0.88
	2.5	37	162	145	0.90
	5.0	38	0	0	n/a
	10.0	40	0	0	n/a
	20.0	40	0	0	n/a
*R. varieornatus* (anh.)	0	41	819	689	0.84
	2.5	40	211	161	0.76
	5.0	41	101	81	0.80
	10.0	40	36	32	0.89
	20.0	19	20	16	0.80

### DNA Damage and Repair

**Figure 3:**
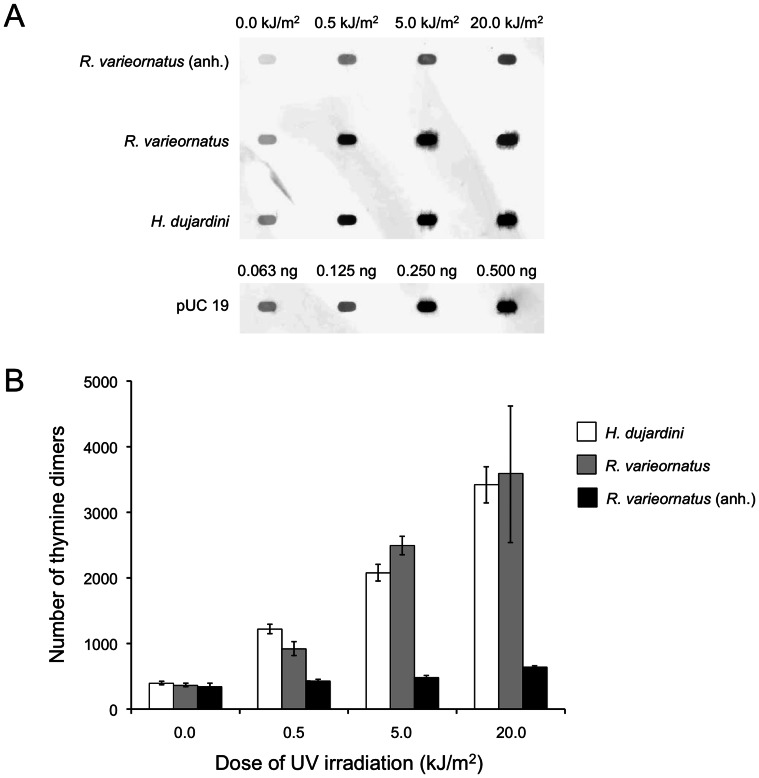
Thymine dimer formation in DNA in tardigrades after UVC irradiation. (A) An image of dot blot analysis showing thymine dimer formation after UVC exposure in *Hypsibius dujardini* and *Ramazzottius varieornatus* in the hydrated state, and *R*. *varieornatus* in the anhydrobiotic state (anh.), and pUC 19 plasmid DNA used as molecular standard. (B) Thymine dimer formation per megabase in DNA in *H*. *dujardini*, *R. varieornatus*, and *R. varieornatus* in the anhydrobiotic state (anh.). Each value is the mean±SD (n = 3).

The frequency of thymine dimer formation in DNA between hydrated *R. varieornatus* and *H. dujardini* irradiated in the hydrated state was comparable (Two-way ANOVA: *P* = 0.75) (Fig. 3AB). There was a clear dose dependent increase in the frequency of thymine dimer formation detected in both the *R. varieornatus* (Nonlinear regression (curve fit) analysis: *r* ^2^ = 0.85) and *H. dujardini* (Nonlinear regression (curve fit) analysis: *r* ^2^ = 0.94) irradiated in the hydrated state. The number of thymine dimers formed increased by 8.7 times in *H. dujardini* and 9.9 times in *R. varieornatus* irradiated in the hydrated state compared with non-irradiated hydrated control after exposure to 20 kJ/m^2^ of UVC radiation (Fig. 3B). On the other hand, only a slight increase (1.8 times) in the number of thymine dimers was observed in *R. varieornatus* irradiated in the anhydrobiotic state in comparison with non-irradiated anhydrobiotic control (Fig. 3B).

**Figure 4:**
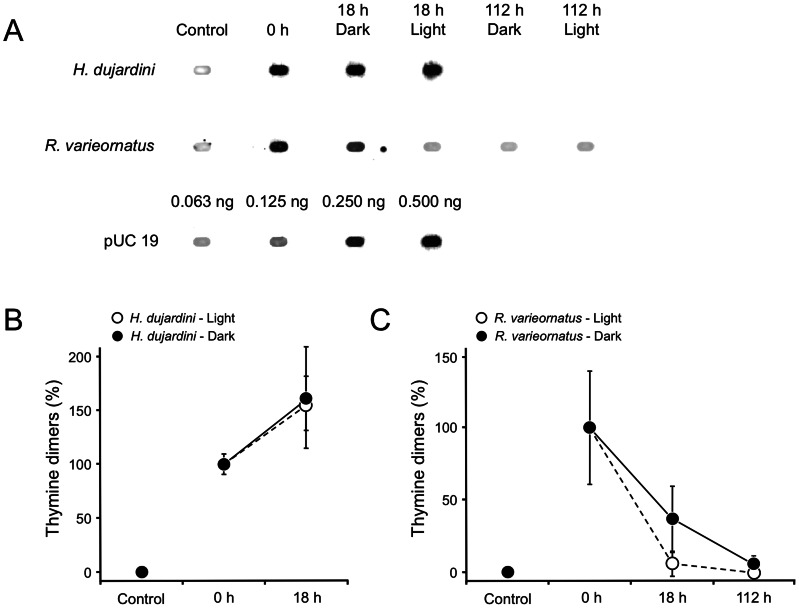
A time course of frequency of thymine dimer formation in DNA in *Hypsibius dujardini* and *Ramazzottius varieornatus* kept under light and dark conditions following UVC irradiation in the hydrated state. (A) A dot blot image showing thymine dimer formation in the two species. (B, C) Percentage of thymine dimers formed following exposure to UVC radiation in *R*. *varieornatus* and *H*. *dujardini* respectively. Control means non-irradiated specimens. pUC 19 plasmid DNA was used as molecular standard. Each value is the mean±SD (n = 3).

We detected DNA repair activity in *R*. *varieornatus* irradiated with 2.5 kJ/m^2^ of UVC radiation in the hydrated state (Fig. 4AB). In hydrated animals of *R. varieornatus,* the level of thymine dimers in DNA was reduced to a similar level in non-irradiated controls in both light and dark conditions at 112 h following UVC irradiation with 2.5 kJ/m^2^. The data proved that *R. varieornatus* has ability to repair DNA lesions inflicted by UVC radiation in the hydrated state within 18 h when exposed to fluorescent light while only approximately 65% of DNA lesions were repaired at this point when the animals were kept under dark conditions. In both light and dark groups of *H. dujardini,* no specimens survived after irradiation, and the number of thymine dimers at 18 h after irradiation increased by approximately 50% compared at the time just after irradiation in both the groups, suggesting that new thymine dimers kept being formed by non-biological chemical reaction in dead *H*. *dujardini*. The spots shown in non-irradiated control specimens (Fig. 3A, Fig. 4A) may have resulted non-specific binding by antibodies used in the experiments.

### Photorepair Gene

**Figure 5:**
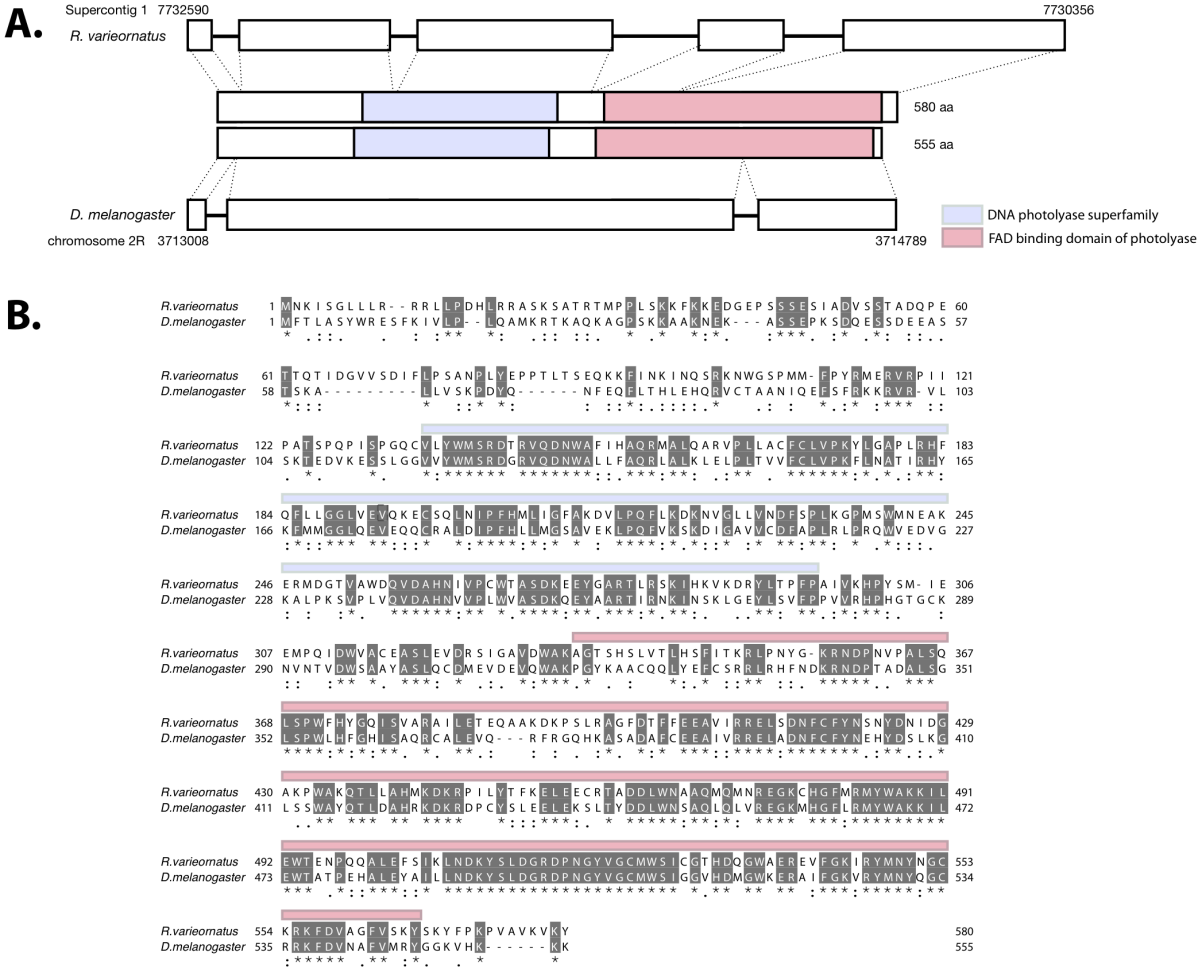
Gene structure of putative *phrA* gene of *R. varieornatus*. (A) Exon-intron structures of *phrA* in *R. varieornatus* and *D. melanogaster*. (B) Amino acid sequence alignment of the *phrA* genes between *R. varieornatus* and *D. melanogaster*.

As DNA repair activity was reinforced by fluorescent light illumination, it was hypothesized that *Ramazzottius varieornatus* perform light dependent DNA repair due to the presence of a photorepair enzyme. The genome of *R. varieornatus* has already been sequenced (unpublished data) but for *H. dujardini,* only an EST library is available (http://www.ncbi.nlm.nih.gov/genomeprj/20353). A putative homolog of the *D. melanogaster phrA* was found in supercontig1 of *R. varieornatus*. According to the genome annotation, this gene contains 4 introns, 5 exons and a protein of 580aa (Fig. 5AB). While the exon-intron structures have diverged, sequence lengths and the two functional domains are well conserved among these species (Fig. 5AB).

PCR of the putative *phrA* gene from *R. varieornatus* produced an amplicon for the gene for all temperature gradients attempted (62–72°C). This amplicon was cloned and sequenced and matched the putative PhrA gene from the *R. varieornatus* genome. The *H. dujardini* template did not produce any amplicons for temperatures ranging from 58 to 72 °C.

Exonerate [17] was used to search for the four exons from the phrA gene found in the *R. varieornatus* genome against the *H. dujardini* EST library (http://www.ncbi.nlm.nih.gov/genomeprj/20353), but no homologous gene was found in the *H. dujardini* EST library.

### Gene Expression during Light and Dark Conditions

**Figure 6:**
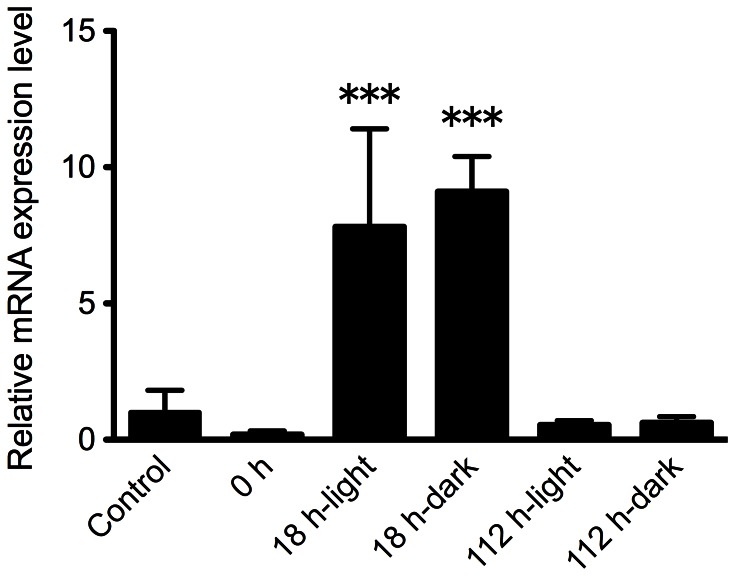
Expression of the *phrA* gene following 2.5 kJ/m^2^ UVC exposure to *R. varieornatus* A value obtained from non-irradiated *R. varieornatus* was set as 1 and values were compared to this control value. Subsequent samples are compared in terms of -fold regulation to this value. Asterisks denote significant differences compared with control (One-way ANOVA; •••: *P*<0.001). Each value is the mean±SD (n = 3).

Expression of the *phrA* gene was evaluated following 2.5 kJ/m2 UVC exposure to *R. varieornatus*. A value obtained from non-irradiated *R. varieornatus* was set as 1 and values were compared to this control value. A significant increase in expression level of *phrA* was observed only 18 h after UVC irradiation in both dark (9.1-fold) and light (7.8-fold) conditions (One-way ANOVA, *P*<0.001) while there was no significant differences in expression levels in 0 and 112 h after UVC exposure compared to that in non-irradiated control (One-way ANOVA, *P*>0.05) (Fig. 6). No significant difference in expression level of *phrA* was detected between dark and light conditions 18 h after UVC irradiation.

## Discussion

Tardigrades are animals well known for their ability to withstand extreme conditions including severe desiccation and extraordinary levels of ionizing and UV radiation [2]–[8]. In the present study we found that *R. varieornatus* had higher survival ability against UVC radiation than *H. dujardini* when exposed in the hydrated state. *R. varieornatus* irradiated with 2.5 kJ/m^2^ of UVC radiation in the hydrated state produced live next generation, indicating that tardigrades are one of the most resistant multicellular organims against UV radiation. To explore mechanisms underlying the survival and reproductive capability to UVC radiation in *R*. *varieornatus* in the hydrated state, we focused on UVC-induced DNA damage and quantified thymine dimer formation in tardigrade DNA following UVC exposure. The frequency of thymine dimers formed in DNA in the hydrated *R. varieornatus* and *H. dujardini* immediately after UVC irradiation was comparable (Two-way ANOVA: *P* = 0.75) (Fig. 3B), although there was remarkable difference in UVC radiation tolerance between the two species.

It is likely that *R*. *varieornatus* does not have a superior ability to avoid forming thymine dimers in DNA compared to *H*. *dujardini*. Rather, as we find for *R*. *varieornatus*, the ability of this species to repair DNA seems to account for its higher UVC radiation tolerance than *H*. *dujardini*. The higher repair activity found in *R. varieornatus* in the light condition suggests that this species has light dependent DNA repair photolyases. The photolyase gene *phrA* homolog found in *R. varieornatus* genome may explain the DNA repair activity shown in this species. PhrA, a CPD DNA photolyase, repairs damaged DNA by reversing CPDs to their monomeric form by nucleotide excision accompanied with subsequent exposure to blue light (< 300 nm) [21], [22]. The *phrA* gene was up-regulated at 18 h after UVC irradiation and expression level of this gene at 112 h post-UVC exposure at which damaged DNA was repaired completely decreased down to comparable levels at non-irradiated control and 0 h after UVC irradiation in both the dark and light conditions (Fig. 4C, Fig. 6). It suggests that PhrA is involved in repairing DNA damage induced by UVC radiation in *R. varieornatus* and that the extraordinary tolerance of *R*. *varieornatus* to UVC radiation is based on the ability to repair UVC-induced DNA damage at least in part dependent on PhrA since pyrimidine dimer formation in DNA prevents DNA replication and could cause organismal or cell death [22], leading to infertility due to failure of development of an embryo.

Desiccation tolerant tardigrades in hydrated state have been shown to survive various kinds of extreme environmental conditions including UV radiation [2]–[8], [23]. UV radiation tolerance shown in *R. varieornatus* in the present study is supposed to be linked to desiccation tolerance, because both radiation and desiccation cause critical protein oxidation [24], [25]. *R*. *varieornatus* showed higher desiccation tolerance than *H*. *dujardini* [Table S1]. There would be efficient systems for protecting proteins against oxidation in *R*. *varieornatus*, accounting for its superior tolerance to UVC radiation compared with *H*. *dujardini*. It was reported that genes encoding antioxidant proteins such as glutathione S-transferase (GST) and superoxide dismutase (SOD) were up-regulated upon desiccation in the tardigrade *H. dujardini, M. tardigradum,* and *R. coronifer* [26] and that an increase in activity of SOD was confirmed in the tardigrade *Paramacrobiotus richtersi* [27]. In addition, recent study [28] suggests that carotenoids identified in the tardigrade *Echiniscus blumi* play a role in protecting the animal against oxidation. Thus, based on these researches, it is expected that *R*. *varieornatus* has prominent systems for protecting its cells from UVC-induced oxidation to sustain normal biological function, including systems for repairing UVC-induced DNA damage, although not studied here. The similar correlation between radiation toleration and desiccation tolerance in rotifer species was reported in previous study [29].

*R. varieornatus* irradiated with UVC radiation in the anhydrobiotic state showed further survival and reproductive capability than that in the hydrated state, indicating that anhydrobiosis made *R. varieornatus* further tolerant to UVC radiation. This tendency was consistent with results by May et al. (1964) [4] showing that the tardigrade *Macrobiotus areolatus* in the anhydrobiotic state survived much longer UV radiation exposure than hydrated animals (the authors did not measure UV radiation doses for their study). The anhydrobiotes accumulated fewer thymine dimers compared with the hydrated animals following exposure to UVC radiation in this study. Riklis (1965) [30] reported that the number of thymine dimers formed in dry DNA was only about 1/10 of that in wet DNA *in vitro*. In *R*. *varieornatus* body water content reduces from 78.6% in the hydrated state to only 2.54% wt/wt in the anhydrobiotic state [3]. Thus, the remarkable UVC radiation tolerance of *R*. *varieornatus* in the anhydrobiotic state could be attributed to the ability to prevent UVC-induced thymine dimer formation in its dehydrated DNA. A similar situation occurs in bacteria. Spores of the bacterium *Bacillus* species are more resistant to UV radiation and accumulate fewer thymine dimers in DNA than growing cells following exposure to UV radiation [31]. Instead, the *Bacillus* spores accumulate detrimental 5-thyminyl-5, 6-dihydrothymine as a major photoproducts induced by UV radiation, and the spores have systems to repair the photoproducts [31].

Collectively, the present study proposes that *R. varieornatus* tolerates UVC radiation by utilizing mechanisms for repairing UVC-induced thymine dimers in DNA in the hydrated state, and by avoiding forming those dimers in the anhydrobiotic state.

## Supporting Information

Figure S1 **The spectrum of the UV radiation emitted by the lamp to irradiate tardigrades.**
(TIF)

Table S1 **Survival of** ***H. dujardini*** **and** ***R. varieornatus*** **24 h after desiccation under 33.8% relative humidity at 22°C for 5 days.**
(DOCX)
