# Supplementary figures and images for: Analysis of DNA Repair and Protection in the Tardigrade Ramazzottius varieornatus and Hypsibius dujardini after Exposure to UVC Radiation
Source: PLoS One. 2013 Jun 6;8(6):e64793. doi: 10.1371/journal.pone.0064793 (PMC3675078; doi:10.1371/journal.pone.0064793)

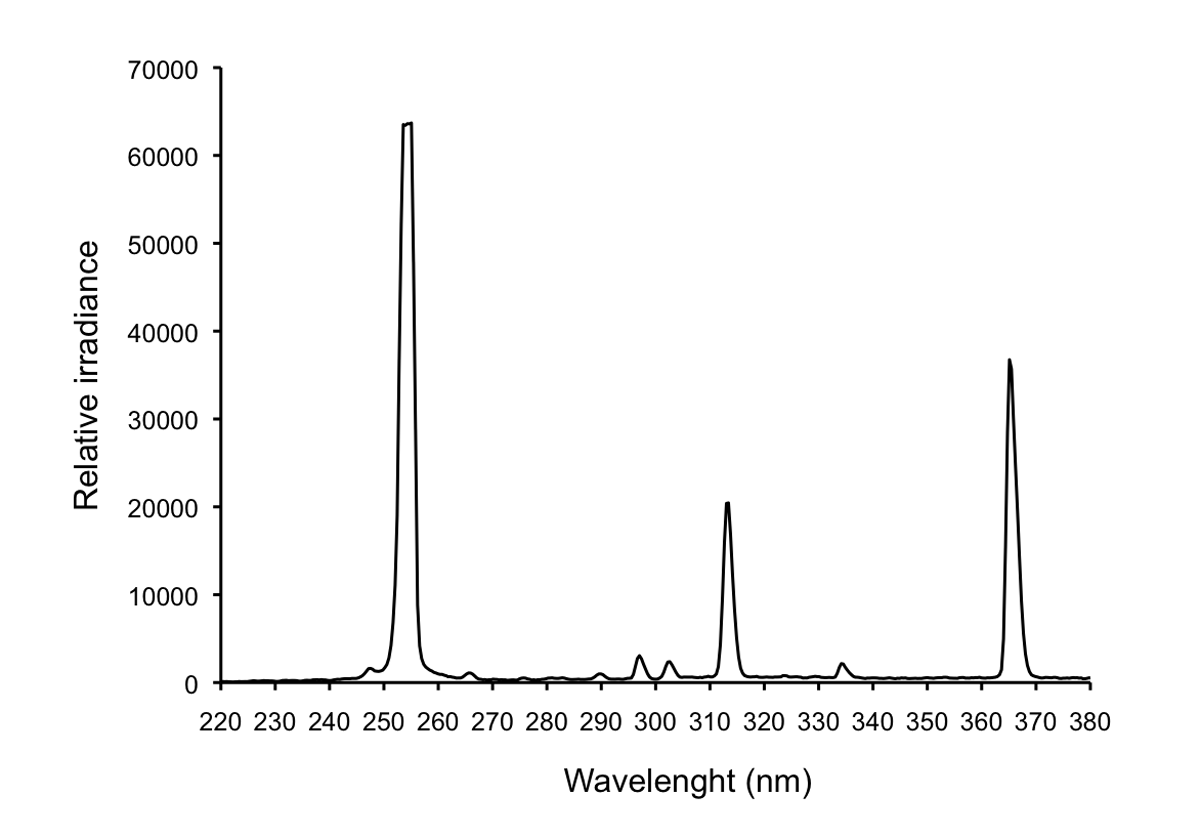

Supplement: Figure S1 — The spectrum of the UV radiation emitted by the lamp to irradiate tardigrades. (TIF) [file pone.0064793.s001.tif]
