# Supplementary material for: Analysis of DNA Repair and Protection in the Tardigrade Ramazzottius varieornatus and Hypsibius dujardini after Exposure to UVC Radiation
Source: PLoS One. 2013 Jun 6;8(6):e64793. doi: 10.1371/journal.pone.0064793 (PMC3675078; doi:10.1371/journal.pone.0064793)
Supplement: Table S1 — Survival of H. dujardini and R. varieornatus 24 h after desiccation under 33.8% relative humidity at 22°C for 5 days. (DOCX) [file pone.0064793.s002.docx]

**Table S1** Survival of *H. dujardini* and *R. varieornatus* 24 h after desiccation under 33.8% relative humidity at 22ºC for 5 days.

| Species | Total specimens, n | Trials, n | Survival, % |
| --- | --- | --- | --- |
| *H. duhardini* | 78 | 4 | 0 |
| *R. varieornatus* | 80 | 4 | 98.8±2.5 |
